# Annotations

**Published:** 1887-04-23

**Authors:** 


### Annotations.

The King of the Belgians at the London Hospital.

The King of the Belgians had a great opportunity for self-improvement the other day, when he visited the London Hospital. That *imperium in imperio* is a model little state under the control of model administrators. There moderate resources are used to the utmost advantage, and produce the most admirable results. If money be wasted in hospitals—and it sometimes is—it is not wasted at the London Hospital. His Majesty inspected many of the wards, and expressed himself highly pleased with the excellent arrangements. The London is a good example of what a hospital can do with moderate means, when it is compelled. The care of the sick of the East End devolves almost entirely upon it. What a huge care that must be it is not difficult to imagine. The poor people in such a district must have help in sickness, whether the resources at command be great or small. Adequate resources, it would perhaps be impossible to obtain. A quarter of a million annually might probably be spent without pauperising anybody. No such sum is at the command of the London Hospital. But those resources which are really available are administered with skill, kindness, and judgment of the highest order Nowhere could his Belgian majesty have witnessed a better example of the adaptation of means to ends, and of the noble energy of the voluntary principle.

Admission and Discharge of Patients.

The Huntingdon County Hospital has just held its adjourned general meeting. According to the old rules no domestic servant "in place" could be admitted as an in-patient. That rule was probably justified by circumstances when it was made, although it must often have proved a source of great hardship to individuals in domestic service. It is now very properly rescinded. A rather lengthened discussion arose about the admission and discharge of ordinary in-patients. The managers have appointed a committee, consisting of not less than two subscribers, whose duty it shall be to examine patients' letters of recommendation, to admit, reject, and discharge patients, etc. Such a committee with such functions is not a very common institution, and it may be hoped that most hospitals will find themselves able to manage the admission and discharge of patients without one. Its creation appears to have resulted from the fact, that a certain patient was discharged without the sanction of the medical practitioner in charge. Who could have taken upon himself to assume such a responsibility? Was it the secretary, or could it possibly have been the matron? We have no means of answering these questions. One thing is quite certain, viz., that no person, except the medical officer in charge, should have anything to do with the *discharge* of in-patients. He alone is practically competent to say when discharge is safe and right; and if there be anything connected with himself personally which unfits him for such responsibility, he should speedily be replaced by a suitable successor. With regard to the *admission* of in-patients, the case is different. Here, also, the doctor alone can advise as to the medical or surgical fitness of all cases; but other questions than these are often involved, in country hospitals especially, and there need be no offence taken by him, if an admission committee help him to decide those other questions. The hospital managers seem to be carefully feeling their way to a judicious method of dealing with the subject. It is not, however, as a rule, desirable, to take the responsibility of admission any more than that of the discharge of in-patients, out of the hands of the medical attendant.

Religion in French Hospitals.

Can it be true, as reported by the *Evening Standard* of the 12th instant, that "a patient in one of the Paris hospitals has written to the Muncipal Council to complain that whilst on a sick-bed he urgently entreated a priest might be sent for, but in vain, the attendance of a minister of religion being refused on one pretext or another. More than this, the muncipal councillors, in considering the incident, passed a vote of congratulation to the lay hospital attendants who had deprived the patient of the religious consolation he asked for?" We do not like to form hasty judgments; but this state of things demands more than passing consideration. What kind of system must that be, which, after hundreds of years of honourable opportunity, has made itself so hated by men of all classes that hospital nurses and municipal councillors will unite in barricading the doors of the hospital against it? Are we to consider that the French are a hopelessly atheistic people, or must we conclude that the Romish Church has so misused its opportunities as to defeat every end for which religious organisations exist? Unless there be some good explanation the action of the hospital officials and of the municipal councillors in refusing to grant the patient the religious ministrations he desired is utterly barbarous and inexcusable. But whilst that is so, the Catholic Church and other religious bodies should remember that meekness, charity, and patience, not rule, authority, and tyranny, are the characteristics which men look for in religion, and by which alone any religion can hold permanent sway in their hearts. Too often in the history of the world the Catholic Church has had itself to thank for the bitter feelings displayed by its enemies. We do not say these things from any mental hostility, but in the interests of that true religion which should be the hope and consolation of all men.

